# Perception and incidence of Buruli ulcer in Ogun State, South West Nigeria: intensive epidemiological survey and public health intervention recommended

**DOI:** 10.11604/pamj.2018.29.166.10110

**Published:** 2018-03-22

**Authors:** Patricia Ihuaku Otuh, Festus Olukayode Soyinka, Bamidele Nyemike Ogunro, Victor Akinseye, Elebe Emmanuel Nwezza, Adebola Olubunmi Iseoluwa-Adelokiki, Olanike Kudirat Adeyemo

**Affiliations:** 1 Department of Veterinary Public Health and Preventive Medicine, University Of Ibadan, Ibadan, Oyo state, Nigeria; 2 Veterinary Teaching Hospital, University of Ibadan, Ibadan, Oyo State, Nigeria; 3 Ogun State Tuberculosis, Leprosy and Buruli Ulcer Control Programme, Ministry of Health, Abeokuta, Ogun State, Nigeria; 4 Department of Mathematics/Computer Science/Statistics and informatics, Federal University Ndufu Alike Ikwo, Ebonyi State, Nigeria

**Keywords:** Buruli ulcer, Mycobacterium ulcerans, Epidemiology, Neglected tropical disease, Rural population, Nigeria

## Abstract

**Introduction:**

Buruli ulcer (BU) is a highly ranked neglected tropical disease (NTD) of global health importance with increasing incidence in sub-Saharan Africa yet there is paucity of information on the epidemiology of BU in Nigeria. Incidentally, highly BU endemic Benin Republic shares proximity with Nigeria. This study was carried out to establish presence of BU and ascertain the level of BU perception among rural populace in Ogun State, south-west Nigeria.

**Methods:**

Secondary data (2009-2012) on incidence of BU was collected from a reference hospital. A cross-sectional survey using structured questionnaire administered to rural people and healthcare practitioners was conducted in three purposively chosen Local Government Areas (LGAs) in Ogun State based on unpublished reports of BU presence.

**Results:**

Data collected revealed 27 hospital confirmed BU cases between 2009-2012 across four LGAs (Obafemi Owode, Abeokuta North, Yewa North and Yewa South) while 14%(21/150) chronic ulcers (suspected to be BU) were discovered during the cross-sectional survey carried out in Odeda, Yewa South and North LGAs. Healthcare practitioners 63.6% (42/66) and 54.7% (82/150) rural people demonstrated poor level of BU perception respectively.

**Conclusion:**

This study provides evidence that BU exists in Ogun State and evaluates the poor perception that the affected rural populace has on the disease. This pilot study presents baseline information on BU in a rural setting in Ogun State South-west Nigeria hence the vital need for prompt public health involvement and further research on the epidemiology of BU.

## Introduction

Buruli ulcer (BU) is an emerging tropical disease predominant in West Africa, affecting mostly rural populace who most times are quite ignorant of the disease. The BU is a very devastating disease of humans and animals caused by an environmental non-tuberculous mycobacterium (NTM), *Mycobacterium ulcerans* [1-5]. Children under the age of fifteen years have been found to be commonly affected by this highly rated neglected tropical disease (NTD) inflicting huge impact inform of deformities, functional limitations and social stigma on them when assessed by disability-adjusted life years, DALYs [6-9]. Increased morbidity due to extensive necrosis of the skin, subcutaneous tissues, bones involvement and varying degrees of contracture on BU patients is very common and compounded with inadequate or no access to health care services in rural settings, all contributing to the lowest possible standard of living among affected people [2,10-13]. The global health concern for BU as revealed by WHO classification of NTD shows that BU is a top ranking emerging NTD [14]. Although BU is noted as the third most common mycobacteriosis, some communities in the sub-Saharan Africa are recording incidence surpassing Tuberculosis and Leprosy [7,10,15-17]. The causative agent *M. ulcerans* has affinity for aquatic areas; rivers, streams, farmlands, irrigated areas, where majority of the rural poor conduct several livelihood activities ranging from farming, fishing to fetching water for domestic purposes [1,10,18-24].

Despite increased geographical spread of Buruli ulcer in sub-Saharan Africa especially in West Africa, there is dearth of information on epidemiology of the disease in Nigeria when compared with some of West African countries: Ghana, Benin Republic, Togo, and Cameroon [7,10,25-27]. Previous reports on BU in Nigeria have always been focused on clinical cases recorded either by hospital presentation or as a result of active case searches [16,28-31]. The epidemiology of BU is very important due to its yet to be unraveled mode of transmission which also hampers efforts to prevent and control the disease [10,20,21,32,33]. Consequently, information on the perception of BU disease among the most predisposed rural populace is very vital in achieving the meaningful goals of finding out the prevalence of BU which is uncertain in most endemic countries, encouraging early reporting by affected people and ensuring prompt treatment thereby preventing the extensive devastating effects of BU [20,34,35]. The objectives of this study were therefore to establish the presence of BU and BU perception by people living in rural susceptible villages in Odeda, Yewa North and Yewa South Local Government Areas of Ogun State which shares close boundary with Benin Republic; a BU epicenter. The outcome of this study serves as evidence based guideline for policy formulation in the health sector of Ogun state and Nigeria at large which should be geared towards implementation and provision of prompt medical assistance to alleviate the anguish suffered by BU patients. In addition this work provides preliminary information for further research into the epidemiology of *Mycobacterium ulcerans* in Nigeria.

## Methods

### Study location

This study was undertaken in Ogun state, one of the six states in the south west geo-political zone of Nigeria. It is bordered by the highly populous Lagos state to the south, Oyo and Osun states to the north, Ondo state to the east and Republic of Benin to the west. It has an estimated population of 3,751,140 according to the 2006 population census and covers a land mass of 16,762 square kilometers. The state is located on the geographical grid reference of 6.90980N and 3.25840E [36]. Three local government areas (Odeda, Yewa South and Yewa North) were purposively selected for this study based on previous unpublished report of BU presence.

### Study design

Secondary data collection was first carried out at the Hansen's disease center Iberekodo, Abeokuta North Local Government Area of Ogun State. Information on BU patients presented to the facility between 2009 and 2012 was retrieved from the hospital record. The number of patients and their corresponding communities of residence were noted. This was followed by a descriptive observational study from January to October 2013 involving cross-sectional survey which adopted the administration of structured questionnaire to consenting respondents in the three local government areas (Odeda, Yewa North and Yewa South). Two categories of respondents were recruited; the rural people comprising adult males and females and the health care practitioners made up of community healthcare workers (CHW), nurses and medical doctors. Information gathered were, socio-demographic variables including local government area, gender, level of education, age, class of health facility and duration on the job. The perception level was based on responses of the respondents to questions relating to awareness on BU, its causative agent, forms of BU presentation, possible ways of exposure, treatability, methods/modes of treatment and attitude towards BU patients. The responses were scored on a scale of 1 to 6 with scores ranging from 1-3 regarded as poor perception and 4-6 as good perception.

### Data analysis

Data were analyzed using SPSS ® (Statistical Package for Social Sciences) version 20. Socio-demographic variables were summarized using descriptive statistics. The Sign-test for non-parametric data was used to analyze the socio demographic predictors of BU perception among the rural people and healthcare practitioners. Significance was set at p ≤ 0.05.

## Results

### Buruli ulcer presence in Ogun State

**Figure 1:**
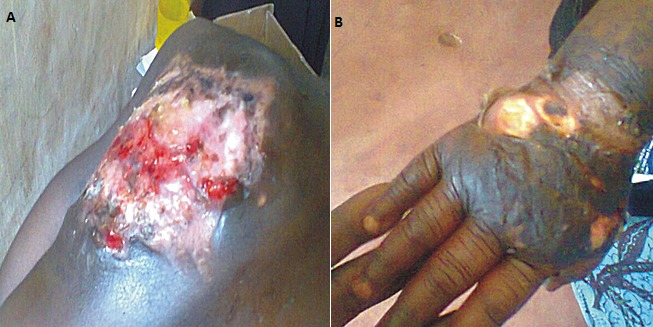
Buruli ulcer cases found during the cross-sectional survey

The hospital record of Hansen's disease center Iberekodo located in Abeokuta North LGA revealed cases of BU presented to the facility between 2009 and 2012. The patients were from four different LGAs; Yewa South, Yewa North, Obafemi Owode and Abeokuta North. A total of 27 BU cases were presented within the 4year period. Of the total number of cases presented; 44.4% (12/27) was in 2009; 22.2% (6/27) in 2010; 3.7% (1/27) in 20^11^ and 29.6% (8/27) in 20^12^. Yewa South LGA had the highest number of BU cases 59.3% in three years (2009, 2010 and 2012) with no case in 2011 while Yewa North LGA with 29.6% of the BU recorded had cases in all the four years. Only one BU case was from Obafemi Owode LGA in 2009 and 2cases were from Abeokuta south LGA in 2012. During the cross-sectional survey conducted in the study location, a total of 21 cases (14%) presenting with varying degrees of unhealed ulcers with close tallying with BU case definition were discovered (Figure 1). Yewa south recorded the highest number (9), followed by Odeda (7) and Yewa north (5). The duration of the ulcers ranged from 1-35years and majority were located on the lower extremity but only one case was found at the back.

### The outcome of cross-sectional survey

A total of one hundred and fifty (150) respondents from the rural people category participated in the study across the three LGAs purposively selected for this study. Odeda, Yewa South and Yewa North LGAs respectively had 33, 59 and 58 respondents. In healthcare practitioners' category 66 participants were involved in the survey. The distribution of the various respondents; community healthcare workers (CHW), nurses and medical doctors were as follows respectively: Odeda LGA-17, 11, and 5; Yewa North LGA-14, 6, 2 and Yewa South LGA-2, 8, 1.

### Buruli ulcer perception

54.7% of the total respondents had poor level of BU perception among the rural people who participated in the survey. However Yewa South LGA had the best BU perception (33/68=48.5%) while Yewa North had the least level of BU perception (37/82=45.1%). More than half of the respondents were males (65.3%) despite this the male respondents had poorer BU perception than the females. 57/98 (58.2%) males had poor perception whereas 27/52(51.9%) of the females had good perception. People with formal education with the exception of tertiary level and the uneducated all had poor level of BU perception. 30% of respondents within the age bracket of 30-39 displayed the poorest BU perception within the age demography. Perception of respondents towards BU indicated significant association with all the demographic predictors as revealed in Table 1. Among the 66 respondents in the healthcare practitioner's category 63.5 % had poor BU perception. In the class of healthcare facility, the secondary healthcare facility workers presented a better level of BU perception than the primary healthcare facility counterpart. Healthcare practitioners working in the primary healthcare facilities had 54.6% of poor perception. The younger healthcare practitioners demonstrated a higher level of good perception than the older practitioners. There is significant association between BU perception and all the socio-demographic variables (LGA, class of health facility and duration on the job) as shown in Table 2.

**Table 1:** Socio-demographic predictors of BU perception among rural people

CATEGORY		Good Perception		Poor Perception		Confidence interval	P-value
		Frequency (n^+^)	%	Frequency (n^+^)	%		
LGA	Odeda	14	9.3	19	12.7	0.000-.020	0.007^++^
	Yewa North	21	14.0	38	25.3		
	Yewa South	33	22.0	26	17.3		
Gender	Male	41	27.3	57	38.0	0.000-.020	0.000^++^
	Female	27	18.0	25	16.7		
Education level	No education	14	9.3	17	11.3	0.000-.020	0.000^++^
	Primary	21	14.0	31	20.7		
	Secondary	21	14.0	24	16.0		
	Tertiary	12	8.0	10	6.7		
Age	20-29	16	10.7	8	5.3	0.000-.020	0.000^++^
	30-39	23	15.3	45	30.0		
	40-49	19	12.7	20	13.3		
	>50	10	6.7	9	6.0		

+ n = number of respondents,

++ = significant

**Table 2:** Socio-demographic predictors of BU perception among healthcare practitioners

CATEGORY		Good Perception		Poor Perception		Confidenceinterval	P-value
		Frequency (n^+^)	%	Frequency (n^+^)	%		
LGA	Odeda	11	16.7	22	33.3	0.000 - 0.049	0.000^++^
	Yewa North	5	7.6	6	9.1		
	Yewa South	8	12.1	14	21.2		
CLASS OF HEALTH FACILIT	Primary	16	24.2	36	54.6	0.000 - 0.049	0.000^++^
	Secondary	8	12.1	6	9.1		
DURATION ON THE JOB(YEARS)	1-5	17	25.8	10	15.2	0.000 - 0.049	0.017^++^
	6-10	2	3.0	8	12.1		
	11-20	2	3.0	12	18.2		
	> 20	3	4.5	12	18.2		

+ n = number of respondents,

++ = significant

## Discussion

Difficulty in unraveling the mode of transmission of *M. ulcerans* the causative agent of Buruli ulcer creates a huge obstacle in proffering actual preventive and control strategies for this debilitating disease ravaging impoverished population in sub-Saharan Africa [37, 38]. To achieve these phenomenal feat, in-depth epidemiological studies on BU is of paramount importance most especially in the endemic West African region [7, 21, 33]. The population at risk has enormous part to play in providing relevant information on the disease which will enable researchers to be well equipped with essential information central for positive findings. This study therefore ascertained the level of acuity of the people inhabiting the locations in Ogun with evidence of having BU patients and which shares close geographical proximity with the highly endemic Benin Republic [26,39]. Our study established the presence and level of BU cases presented to the Hansen's disease centre; a referral facility which provided an insight on the probable locations for further epidemiological investigation, this is in line with work carried out by Taban et *al*.; Owusu and Adamba [7, 37] in Cameroon and Ghana respectively who employed cross-sectional studies on the basis of retrospective studies in BU health district facilities. Our study revealed the trend and annual distribution of BU disease in Ogun state from 2009-2012 bringing to the fore that the highest BU cases were most prevalent (59.3%) in Yewa South. According to the hospital reports BU case search was conducted proceeding this period which must have enlightened the rural people to understand the importance of seeking medical help. Several studies involving active BU case search have been successful in Nigeria and in some African countries towards epidemiology and molecular studies of BU disease [16, 24, 29, 30]. The focal distribution of BU was also highlighted in this study which is in line with findings from previous reports from some West African countries [38, 40]. The cross-sectional survey involving questionnaire administration yielded results from the study location with the highest number of BU patients emanating from Yewa South LGA hence tallying with the report from the hospital records. This may not be unconnected with the close proximity that this LGA shares with highly endemic Benin Republic. Reports have it that many BU patients from Nigeria access medical help from neighbouring countries [40]. Odeda LGA had no previous BU report but are susceptible on account of the predisposing risk factor of culpable aquatic environments situated within the communities. There is there possibility of Odeda becoming new BU foci. Majority of the ulcers were located on the lower extremity in accordance with previous reports [16, 32] attributing this to probably the ease of contact with risk factors.

People living in Yewa South and Yewa North LGAs were more willing to participate in the survey perhaps because there were BU patients within these localities. However in the healthcare practitioner category, more well-disposed respondents were encountered in Odeda LGA. The closeness of this LGA to Ibadan; a peri-urban city must have been the reason associated to this, as healthcare service delivery seems more accessible. This study equally highlighted increased number of CHCW revealing their impact on rural health care delivery thereby affirming previous studies carried out in Republic of Benin and in sub-Saharan Africa [41-43]. There was generalised evidence of poor perception across the study area, pointing to the high level of ignorance on BU among the rural populace. Majority attributed occurrence of BU to witchcraft, spiritual attack, ancestral curse and affliction by *"ofa"* hence the believe that only appeasing the gods, certain sacrifices, traditional healing and deliverances can remedy BU. The availability of free BU treatment opportunity was only known by few healthcare practitioners and some BU patients who had received prior treatment. Women were more knowledgeable, most likely because they bear most of the burden suffered by BU patients directly [43-45]. Although CHCW contributed immensely to the care of BU patients they still had poor perception on BU most likely because of their low level of education. From this study, education of both rural populace and healthcare providers cannot be over emphasized.

## Conclusion

Globally Buruli ulcer disease has assumed a great health concern because of the severe pain, disfigurement and harsh economic burden on affected people. Efforts are on to discover the exact mode of transmission, extent of spread through geographical mapping, rapid diagnostic techniques which will easily be accessible in the rural areas and for more national sustainable surveillance. Population studies are very important in understanding peculiar diseases inherent in a population. Information on such diseases from the affected people offers very important direction mostly to researchers. Ascertaining the perception of people from this study on BU has provided a vital overview on the knowledge of BU exhibited by these communities; which were fundamentally wrong. Majority of the people affected did not believe that BU is treatable; many believed that witchcraft was the cause and many more did not know that treatment was free. This study has thrown more light on these grey areas and will subsequently spur many to access medical help early to forestall extensive consequences. It is therefore recommended that the Government of Ogun state should increase efforts to strengthen the public health department to undertake intensive and extensive epidemiological studies on BU in all LGAs of the state as this will help in establishing the prevalence of BU in the state. The federal ministry of health equally should have an extended plan of conducting national survey on BU across Nigeria to ascertain the status of Buruli ulcer in Nigeria.

### What is known about this topic

- Buruli ulcer is a neglected tropical disease highly endemic in West Africa;
- Close proximity to aquatic environment is a risk factor.

### What this study adds

- This study provides probable level of BU endemicity and the level of BU perception by the population (rural populace) highly at risk in Ogun state;
- It also promotes essential aspect of increasing awareness on BU especially among the population at risk.

## Competing interests

Authors declare no competing interests.
